# Guide to dynamic OCT data analysis

**DOI:** 10.1364/BOE.571394

**Published:** 2025-10-31

**Authors:** Noah Heldt, Tual Monfort, Rion Morishita, Robert Schönherr, Olivier Thouvenin, Ibrahim Abd El-Sadek, Peter König, Gereon Hüttmann, Kate Grieve, Yoshiaki Yasuno

**Affiliations:** 1 Institute of Biomedical Optics, Universität zu Lübeck, 4 Peter-Monnik-Weg, Lübeck 23562, Germany; 2 German Center of Lung Research (DZL), 130 Aulweg, Gießen 35392, Germany; 3 Sorbonne Université, INSERM, CNRS, Institut de la Vision, 17 rue Moreau, F-75012 Paris, France; 4 Computational Optics Group, University of Tsukuba, 305-8573 Ibaraki, Tsukuba, Japan; 5 Institute of Anatomy, Universität zu Lübeck, 160 Ratzeburger Allee, Lübeck 23562, Germany; 6 Institut Langevin, ESPCI Paris, Université PSL, CNRS, 75005 Paris, France; 7 Department of Physics, Faculty of Science, Damietta University, New Damietta City, Damietta 34517, Egypt

## Abstract

Dynamic optical coherence tomography (DOCT) enhances conventional OCT by providing specific information related to flow dynamics, cell motility, and organelle metabolic activity. These biological phenomena can be detected with varying sensitivity depending on the OCT architecture parameters, including wavelength, numerical aperture, and implementation method (time domain or Fourier domain). Despite its potential, the field lacks standardization as various research groups have independently developed algorithms for specific applications. In this paper, we compare four widely used DOCT algorithms, each employing a distinct analytical approach: power spectral density moment analysis, frequency band visualization, logarithmic intensity variation evaluation, and motility-based analysis. These algorithms were originally optimized for different OCT technologies (full-field OCT, microscopic OCT, swept-source OCT, and spectral domain OCT), which vary in temporal and spatial resolution as well as susceptibility to motion artifacts. To conduct a fair evaluation, we perform comprehensive cross-wise comparisons using datasets acquired from each of these setups. Our findings reveal that each method exhibits unique advantages in specific imaging environments, thereby providing valuable guidance for algorithm selection based on particular application requirements.

## 1. Introduction

While optical coherence tomography (OCT) [1] is a wide-spread imaging technology in the medical field [2], especially in ophthalmology [3,4], its deployment in biology [5,6] remains limited. This underutilization is unwarranted, as OCT possesses remarkable capabilities. Indeed, OCT can acquire label-free and non-invasive images in optically thick tissues, many times the mean free path of light, reaching > 1 mm in some applications [7,8], at faster volume rates than any fluorescence technique [9], and has optical sectioning ability, making it a powerful microscopy approach for biological investigations.

Contrast in OCT is generated by the interference of light backscattered by the sample with a reference field, which provides time gating, amplification, and phase sensitivity. This mechanism enables the detection of key biological features such as extracellular matrix organization [10], myelinated axons [11], filopodia and euchromatin structures [12], and actin structures [13]. By leveraging the coherence properties of interfering light, the axial resolution in OCT can be independent of the system’s numerical aperture (NA) and depth of field, with the potential to exceed the diffraction limit, a characteristic otherwise found only in light sheet microscopy [14]. Consequently, a primary advantage of OCT is its ability to rapidly acquire large fields of view without compromising axial resolution. OCT can probe axial structures from the submicrometer regime across millimeter ranges, thanks to infrared light utilization and interferometric amplification [15]. Regarding transverse resolution, OCT exhibits confocal-like performance [16], reaching a few hundred nanometers [17] when operated with high NA objectives. Moreover, in its full-field implementation, aberrations do not impact spatial resolution but simply diminish the signal-to-noise ratio, thanks to the use of spatially incoherent illumination [18].

The recent development of dynamic OCT (DOCT) [19] represents a significant advancement, enabling the detection of individual active cells and quantification of physiological activity within optically thick, scattering tissues [13,20]. In DOCT, a time series of OCT images is acquired at a constant sample position. The technique exploits interferometric signal fluctuations originating from scatterers’ axial movements, detected through OCT’s phase sensitivity [21], to highlight dynamic structures over static ones that typically backscatter more light. Through this phase sensitivity, DOCT characterizes tissue components according to sub-resolution scatterer dynamics.

While various biological mechanisms can generate interferometric signal fluctuations, DOCT signals have been shown to correlate strongly with cellular ATP production and utilization [13,22,23], as well as with apoptotic live/dead markers [20] and metabolism-related indicators such as the redox ratio measured by nicotinamide adenine dinucleotide (phosphate) (NAD(P)H) and flavin adenine dinucleotide (FAD) excitation [20,24]. The DOCT signal has enabled monitoring of chemical and mechanical stress responses [24,25], identification of tumor cells within tissues through their enhanced dynamic signatures, and assessment of anti-cancer drug efficacy [26]. This functional extension of OCT thus addresses the traditional limitations of specificity while preserving the inherent advantages of label-free, high-speed volumetric imaging.

As a newly emerging imaging modality, DOCT was pioneered by several research groups in parallel, each developing different experimental configurations and algorithms to quantify and visualize the dynamic information retrieved. Due to the complexity of biological data, the wide variety of sample properties, and the varying OCT implementations, multiple approaches have emerged to analyze and represent these complex fluctuations to extract relevant information.

The foundations of DOCT can be traced back to 2004 and 2006, when depth-resolved holographic imaging of dynamic speckle in tumor spheroids was demonstrated [27] and temporal information from OCT time series was first used for angiography applications [28,29]. These approaches used motion contrast to analyze cells and to visualize blood vessels without contrast agents, demonstrating that dynamic information in OCT could provide functional insights beyond structural imaging. A quantification of diffusive processes by OCT was reported in 2010 by Kalkman *et al.* [30].

A critical breakthrough in the development of DOCT approaches came in 2012 with the publication of "Dynamic light scattering optical coherence tomography" by Lee *et al.*[31]. This pioneering work formally integrated dynamic light scattering (DLS) principles with OCT to enable high-resolution 3D imaging of heterogeneous diffusion and flow. The authors developed a theoretical framework that accounted for static and moving particles within the OCT resolution volume, allowing for the estimation of dynamic parameters including axial and transverse velocities and diffusion coefficients. This work provided a robust mathematical foundation for extracting dynamic information from OCT signals and demonstrated applications in imaging the living animal brain. Earlier work by Farhat *et al.* in 2011 [32] had already shown the potential of DLS-OCT for detecting apoptosis, highlighting how temporal fluctuations in OCT signals could reveal cellular processes beyond simple flow measurements.

Another significant advancement came in 2013 with Oldenburg’s work on "Motility-, autocorrelation-, and polarization-sensitive optical coherence tomography" [33], which demonstrated how multiple functional OCT modalities could be combined to differentiate between different cellular components within 3D tissue cultures. Using spectral-domain OCT with axial and lateral in-water resolutions of 3 and 10 μm, respectively, this approach was further refined in 2015 [34] with a modified standard deviation metric designed to be SNR-independent. This innovation, addressed the non-homogeneous SNR characteristic of SD-OCT across the depth of field. It was demonstrated that intracellular motility follows an inverse power law distribution, distinctly different from the Lorentzian distributions expected for diffusive processes. This inverse power law, also known as 1/f noise, is scale invariant, emerges in systems with long-range correlations or memory, and is characterized by the parameters *α* and $R^{2}$ .

In 2016, Apelian *et al.* formally introduced the "dynamic" term in OCT [13], replacing motility- and autocorrelation-based metrics with statistical distribution measures such as standard deviation, which significantly reduced computation time. The following year, Thouvenin *et al.* introduced pixelwise Fourier transform analysis [5], yielding information about the frequencies of signal fluctuations created by scatterer movements within individual voxels. To compress this high-dimensional frequency space into perceivable RGB images, they implemented frequency channel binning, where three frequency ranges were selected and summed per pixel (px) to represent blue, green, and red channels, with blue typically corresponding to slow changes and red to fast changes. Scholler’s introduction of HSB (Hue-Saturation-Brightness) space representation [35] offered an optimized approach that was widely adopted thereafter. In this method, hue is determined by the pixel’s mean frequency, saturation by the standard deviation of the mean frequency peak, and brightness by the OCT amplitude. This approach created more independent channels directly linked to sample properties. More recently, the rolling-phase approach was proposed by Monfort *et al.* [36] to further refine these approaches.

While in the beginning DOCT imaging with subcellular resolution was limited to full-field time domain OCT (FFOCT), a further important step was the demonstration of dynamic contrast in μOCT [37] and microscopic OCT (mOCT) [38], which utilize supercontinuum light sources and high NA objectives. Advanced algorithms were introduced to correct for motion induced noise, which can be especially disturbing in scanned OCT. This includes clustering in the frequency space to automatically identify optimal cut-off frequencies, eliminating the need for arbitrary frequency binning, and potentially revealing more biologically relevant signal patterns [39,40]. These optimizations are introduced to a wide number of samples in this paper. Recently, dynamic contrast was introduced to line-field OCT working at a frame rate of up to 4 kHz [41]. The high B-scan rate allowed to evaluate the dynamic signal based on a series of fast-measured volumes, opening avenues for an effective 3-dimensional registration of the volumes which is needed for *in vivo* imaging.

In 2020, Abd El-Sadek *et al.* developed two quantitative dynamic OCT algorithms, including logarithmic intensity variance (LIV) and OCT correlation decay speed (OCDS) [42]. The proposed methods successfully visualized and quantified the longitudinal necrotic process and drug response of tumor spheroids. In the following year, they developed a 3D DOCT scanning protocol that captured volumetric DOCT signals in 52.4 s [43]. By considering the dependency of LIV values on the size of the acquisition time window, they recently developed two further advanced algorithms, so called authentic LIV (aLIV) and swiftness, enabling a more direct interpretation of DOCT signals [44]. It has further expanded the toolbox of DOCT analysis methods, demonstrating the rich diversity of approaches that continue to evolve in this rapidly developing field.

These various technical implementations of DOCT reflect the growing interest in functional extensions of OCT that can provide metabolic contrast without exogenous labels, opening new avenues for biomedical research and clinical applications. However, the diversity of processing algorithms, developed on different experimental OCT systems, makes DOCT results difficult to compare and interpret across platforms. While comparisons of different DOCT algorithms like [45] already exist, the field is so rapidly evolving that novel state-of-the-art algorithms have not yet been bench-marked, leaving potential users unsure about their capabilities. Further, it is crucial to compare the algorithms not only on the same data but also on a variety of it, captured with different systems and parameters, in order to discern the respective contributions of experimental design from effects of the processing itself. This is something that has not yet been addressed in the literature. Hence, the aim of this paper is to conduct a qualitative comparison of the main DOCT algorithms in an effort to harmonize data representation and enable the community to further develop DOCT towards greater interoperability and interpretability. To this end, we took previously published data of different tissue types and analyzed these by four different algorithms. The datasets were imaged with FFOCT, mOCT and swept-source OCT, using optimized acquisition parameters for the respective samples and imaging systems. The datasets were then exchanged between the groups and processed with their respective algorithm, enabling the investigation of the differences of the algorithms output parameters and the dependency of each algorithm on the imaging parameters of the different OCT setups.

## 2. Imaging systems & data acquisition

### 2.1. Dynamic FFOCT

The Paris OCT system employs a full-field time domain (FFOCT) setup. It consists of a temporally and spatially incoherent light source at 810 nm LED, M810L3, (Thorlabs, Newport, NJ, USA), with a 25 nm full width at half maximum spectral bandwidth, corresponding to a temporal coherence length of 19.73 μm. However, in this dynamic FFOCT (DFFOCT) implementation [17], the axial resolution is dominated by the NA with the objective [46] rather than the spectral bandwidth, corresponding in our case to an axial resolution of 735 nm and a transverse resolution of 386 nm, with an objective of NA = 1.05 (UPLSAPO30XSIR, Olympus, Japan). *En face* interferograms are acquired with a high-speed, high full-well capacity camera (Q-2HFW, Adimec, The Netherlands) enabling a photon shot-noise sensitivity of 68 dB and 1440×1440px acquisition up to 549 fps. Axial scanning is performed using a microscope scanning stage (IX83, Olympus, Japan), coupled to synchronized movements of the reference arm. Retinal explant data were acquired with the system described in [17] coupled to a commercial microscope.

The data acquisition protocol involved 512 frames acquired at 100 Hz, with a time exposure of 1.4 ms, to generate one dynamic color image, resulting in an *en face* acquisition time of 5.12 s. We note that in the most recent publications on DFFOCT, data are acquired at 400 Hz, then binned by a factor of 4 and recast into a 100 Hz data set for improved SNR [17].

### 2.2. Spectral domain microscopic OCT

The Lübeck OCT system is a scanning spectral domain setup, which is described in detail in [38,47]. This microscopic OCT (mOCT) consists of a broadband supercontinuum light source (SuperKExtreme EXW-4 OCT, NKT Photonics, Denmark), from which the spectrum between 550 nm and 950 nm is used, and a 2048 px spectrometer (custom-build, Thorlabs, Germany) with 400 nm bandwidth. This enables an axial resolution of 1.2 μm and an A-scan depth range of about 600 μm in tissue (refractive index 1.35) at frame rates of up to 250 kHz. Samples are scanned by galvanometric scanners (6210h, Cambridge Technologies, USA). To achieve a high lateral resolution of about 1 μm, a 0.3 NA microscope objective (HCX APO L 10x/0.3 WUVI, Leica Microsystems, Germany) is employed.

Data acquisition followed the following protocol: 150 B-Scan frames, composed of 500 A-Scans and 1024 axial points each, were captured with an A-scan rate of 100 kHz. This resulted in a frame repetition time of about 9 ms corresponding to a frame rate of about 110 Hz.

### 2.3. Swept source OCT system and data acquisition

The Tsukuba OCT system is a Jones-matrix swept-source OCT (JM-OCT) setup. The light source is a microelectromechanical system (MEMS) based swept source (AXP50124-8, Axun Technologies, MA) with a central wavelength of 1,310 nm and an A-line rate of 50 kHz. A scanning lens (LSM03, Thorlabs Inc., NJ) with an effective focal length of 36 mm was used. The lateral resolution is 18.1 μm, while the axial resolution (in tissue) was 14 μm. The axial pixel size in tissue is 7.24 μm. The complete specification of the OCT system is published elsewhere [48,49]. Although the JM-OCT device is polarization-sensitive, only polarization-insensitive OCT images, which are the average of four OCT intensity images acquired through four polarization channels, were used for the DOCT imaging.

For 3D DOCT imaging, the lateral field of view is divided into eight subfields (denoted blocks). Each block comprises 16 B-scan locations, and was scanned by quick raster scanning 32 times in 6.5 s. This resulted in 32 repeated frames at each B-scan location and a frame repetition time of 204.8 ms, equivalent to a frame rate of about 4.9 Hz. Using this scanning configuration, a 3D DOCT volume comprising 4,096 frames captured at 128 B-scan locations is obtained in 52.4 s. The lateral scanning area for the samples was 1 × 1 mm^2^. More information on the 3D DOCT scanning protocol is published elsewhere [43].

## 3. Sample preparation

All datasets presented here are already published in the respective papers cited. No new datasets were prepared for this study.

### 3.1. Retina explant

Adult macaque (Macaca fascicularis) retinas [36] were ethically obtained from terminally anesthetized subjects used in unrelated studies, adhering to the French Ministry of Education, Higher Education and Research, as well as NIH, and EU guidelines (2010/63/EU). Post-enucleation, 1 cm^2^ retinal samples were embedded in 1 % low-melting agarose with Neurobasal-A medium (10888022, ThermoFisher) containing 2 M of L-glutamine (G3126, Merck), and sectioned then into 100 μm transverse slices using a vibrating microtome (Leica, Wetzlar), and prepared for imaging in a glass-bottom plate.

### 3.2. Trachea & tongue

Mouse trachea and tongue were obtained from C57BL/6 mice. The trachea was cut open and, like the tongue, pinned onto a silicone coated Petri dish, which was filled with HEPES buffered Ringer solution, as described in detail in [38,47].

### 3.3. Tumor spheroid

Human breast adenocarcinoma (MCF-7 cell-line) purchased from the Japanese Collection of Research Bioresources (JCRB) cell bank was used to form MCF-7 spheroids. On the first day of cell culture, 1,000 cells were seeded in each well of an ultra-low-attachment 96-well plate as explained in detail in [26]. The cells were kept in the cultivation environment at a temperature of 37 ^∘^C and 5 % CO_2_. On the fifth day of cultivation, Paclitaxel (PTX; Taxol) with a concentration of 1 μM was administered to three spheroids for 1, 3, and 6 days. Non-treated spheroids were kept as a control.

## 4. Algorithms

All described algorithms are available at [57].

### 4.1. HSB visualization of power spectral density moments

Three metrics are classically calculated from intensity fluctuations in FFOCT by the Paris group and are described in [35,36]. The first one is the recently introduced Phase Fluctuation Index (PFI), namely <|ΔI(t)|> [36], which aims to emphasize the magnitude of the local phase fluctuations. This metric is advantageously almost linearly linked to both scatterer reflectivity and to the distribution of phase shift variability induced by their active transport, and is independent of the initial phase bias of the scatterers, mitigating artifacts such as speckle and fringes for dynamic structures with low range drift. A high PFI value may either be a strong scatterer moving a little, or a weak scatterer moving a lot. The other two metrics are the mean frequency of the power spectral density (PSD), <PSD>, and the standard deviation of the PSD, StD(PSD), introduced in [35]. The mean frequency is related to the speed of active transport in cells [23], in an attempt to quantify cell activity and motility. However, it can still pick up drifting extracellular material. The standard deviation was implemented to differentiate directional transport from Brownian behavior, respectively quantifying the notions of cell activity/motility and random movement, that may be drifting material for example. In this way, dynamic magnitude is quantified through PFI, quantitative speed through <PSD>, and motility versus Brownian behavior through StD(PSD). Note that the zeroth frequency is excluded from the PSD calculation, since time-domain FFOCT does not allow elimination of background intensity scattered from all depths without phase modulation. The three metrics are then assigned in a Hue-Saturation-Brightness (HSB) color space, with <PSD> allocated to the hue (a cold blue color meaning a slow speed, a hot red color meaning a fast speed), StD(PSD) allocated to the saturation (Brownian motion/white noise being transparent/unsaturated and a very directive transport being saturated), and <|ΔI(t)|> allocated to the brightness channel. Thresholds were established using percentile cut-off values of the distribution: 0.1 and 99.9 %, 5 %, and 99.9 % for the hue, saturation, and brightness channels respectively. We note that <PSD> and StD(PSD) are inverted when input to the HSB space, and so is their display in grayscale throughout the figures in this paper.

For mOCT B-scan data (Figs. 2–3), the brightness channel was displayed on a decimal logarithmic scale to compensate for the SNR decrease at different depths, which differs from the approach used in the *en face* methodology.

The whole process can be calculated in a vectorized fashion, and can therefore be implemented efficiently on GPU. All of the metrics were calculated with MATLAB.

The HSB visualization of PSD moments was calculated for all samples by Tual Monfort. The respective laptop utilized an i7-13850HX (2.1 GHz) (Intel), 64 GB of RAM ThinkPad (Lenovo), a PCIe 4.0 x4 (Lenovo), and an RTX 3500 Ada Generation Laptop GPU (NVIDIA) with 23.04 TFLOPS.

### 4.2. RGB frequency binning

The algorithm used in Lübeck assumes that the signal fluctuations within the sample are stationary and while they may exhibit broad spectral footprints, they can be approximately separated into static, slow, and fast processes. To gain access to these signal fluctuations, the absolute values of the pixel-wise Fourier transformation over time are leveraged. This is computed on linear scaled OCT intensities. However, as the result is of high dimensionality, it is binned to three channels, to form an easily perceivable RGB image. Here the zero frequency amplitude alone is used as the pixel value for the blue channel, as its intensity is several magnitudes larger than the rest of the spectrum. This is the static information across the time series which corresponds to the mean intensity. To automatically find the cut-off frequency between the green and red channel, we sum the pixel-wise spectra, remove the DC frequency and employ the neural gas algorithm [50]. It is run for ten epochs, with an initial learning rate of 0.1, an end learning rate of 0.001, an initial neighborhood radius of 0.1, and an end neighborhood radius of 0.01. This yields two clusters with the cut-off frequency centered between them. Subsequently, for each pixel, all amplitudes above the zero frequency up to and including this cut-off frequency are summed to form the green channel for that pixel. The information thus corresponds to signal fluctuations of medium speed. Similarly all the values above the cut-off frequency are summed to form the red channel, corresponding to rapid signal fluctuations, up to the Nyquist frequency, given by the imaging frame rate. The higher the amplitudes of the frequency bands in a pixel are, the higher the values of their respective channels will be as well.

After binning, the values of each of the three channels are clipped to the channels bottom 0.1 % and top 0.01 % to enhance the contrast. Afterwards, the channels are individually rescaled to the range [0, 1]. This is needed as otherwise the blue channel’s amplitude still remains magnitudes higher than the values of the other channels, resulting in an almost purely blue image.

To further enhance the contrast, especially for image regions with low signal, a histogram matching is performed. In this step, each channel is matched onto a target histogram. As a target we are using the histogram of the logarithmic standard deviation computed over the time axis of the linear OCT intensities, $Log_{10}(StD(I(t)))$ . Lastly the image contrast was enhanced using ImageJ’s automatic contrast/brightness enhancement function.

The computation is rather fast, due to being based on the fast Fourier transformation. The subsequent binning and normalizations add little additional computational needs.

**Fig. 1.**
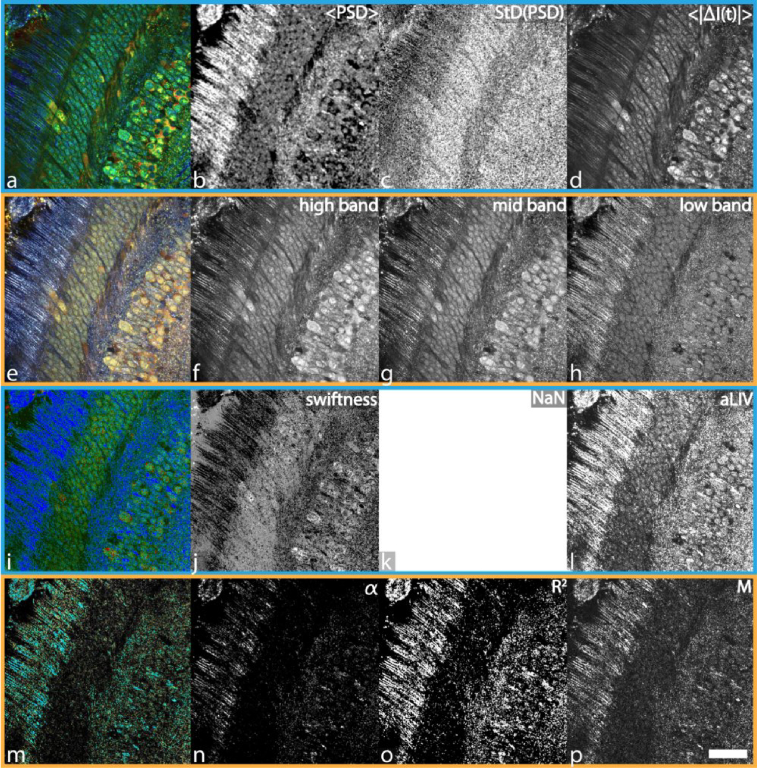
Comparison of the different DOCT algorithms on a macaque retinal explant acquired with the FFOCT. The HSB visualization of the PSD moments was computed in 2.7 s. <PSD> ranges from 2.91 to 14.21 Hz, StD(PSD) ranges from 6.01 to 8.79 Hz, and <|ΔI(t)|> ranges from 0 to 31.18 dB. The adjusted RGB frequency binning was calculated in 14.76 s. The blue channel was determined with frequencies ranging from > 0.1 to 5.96 Hz, the green channel from > 5.96 to 14.36 Hz, and the red channel > 14.36 Hz. The LIV-based aLIV and Swiftness were computed simultaneously in around 46.8 s. Color image (i) was generated with Swiftness (j) ranging from 0.4 to 1.5 s^−1^ for hue (blue-green-red order) and aLIV (l) ranging from 0 to 10^−5^ dB^2^ for brightness. The motility-based metrics took about 22.15 h to compute. Alpha ranges from 1 to 4.71, $R^{2}$ from 0 to 0.99, and M from 0 to 0.13. Color bars can be found in Supplement 1, Table S1. Scale bar: 38 μm. The full resolution figure can be found in Visualization 1.

To process the DFFOCT data (Fig. 1), the algorithm had to be slightly modified however, due to the nature of the DFFOCT acquisition, which does not use a phase modulation in the reference arm for this data. Hence, the DC part of the Fourier transform contains the huge background light scattered from all depths ( Supplement 1, Fig. S1), instead of the OCT signals corresponding to the static structures in the *en face* image. Therefore, the binning method where the zero frequency is used as the blue channel is not suitable. Instead, the first frequency bin was discarded and a neural gas clustering with 3 clusters but otherwise identical parameters was applied.

The RGB frequency binning was calculated for all samples by Noah Heldt. The respective computer utilized an i9-7900X CPU (3.3 GHz) (Intel), 128 GB of RAM (2.133 GHz), a WD_Black SN850X 8 TB M.2 SSD (Western Digital), and a GeForce GTX 1080 Ti GPU (NVIDIA).

### 4.3. Logarithmic-intensity-variance-based algorithm

The algorithm used in Tsukuba is based on logarithmic intensity variance (LIV), which is defined as the time variance of the dB-scaled OCT intensity [42]. Two contrasts, authentic LIV (aLIV) and Swiftness [44], were simultaneously obtained by leveraging the time-window size dependency of LIV. Specifically, multiple LIV values are calculated from data subsets with varying time-window sizes. The LIV value is plotted as a function of the time window and fitted with a first-order saturation function. The aLIV and Swiftness metrics are defined based on the two fitting parameters: the saturation level and the inverse of the time constant, respectively. In principle, LIV and aLIV represent the magnitude of OCT signal fluctuation, while Swiftness represents the speed of the signal fluctuation. Therefore, a high change in magnitudes yields high values in LIV and aLIV, while high values in Swiftness occur by rapid changes. In addition, according to a previous study by the authors [44], aLIV and Swiftness are sensitive to the occupancy and speed of moving scatterers, respectively.

Function fitting of LIV values, which is a computational bottleneck, has been accelerated by using a GPU-based fitting function [44]. Thus, the most time-consuming part in the LIV-based algorithm is the computation of multiple LIV values for several time windows.

**Fig. 2.**
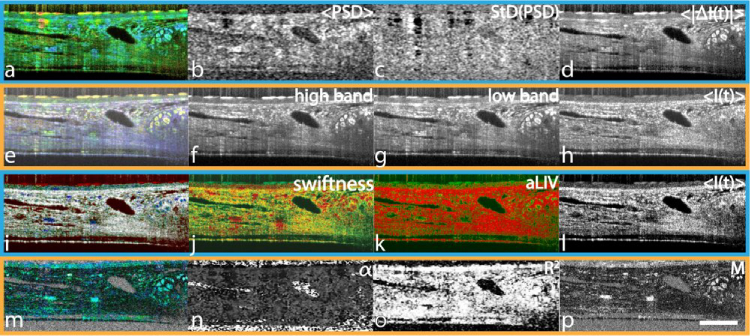
Comparison of the different DOCT algorithms on a mouse trachea explant acquired with the mOCT. The HSB visualization of the PSD moments was computed in 0.44 s. <PSD> ranges from 2.35 to 19.79 Hz, StD(PDS) ranges from 6.21 to 11.36 Hz, and <|ΔI(t)|> ranges from 5.62 to 35.5 dB. The RGB frequency binning was calculated in 1.70 s. The blue channel was determined as the static bin with frequencies < 0.36 Hz, the green channel ranging from > 0.36 to 19.82 Hz, and the red channel > 19.82 Hz. The LIV-based aLIV and Swiftness were computed simultaneously in around 2.73 min. Color image (i) was generated with Swiftness (j) ranging from 10 to 60 s^−1^ for hue (blue-green-red order), aLIV (k) ranging from 0 to 5 dB^2^ for saturation, and OCT intensity (l) ranging from 5 to 25 dB for brightness. The motility-based metrics were computed in 5.47 h. Alpha ranges from 0 to 2.09, $R^{2}$ ranges from 0 to 0.98, and M from 0 to 0.64. Color bars can be found in Supplement 1, Table S1. Scale bar: 100 μm. The full resolution figure can be found in Visualization 2.

**Fig. 3.**
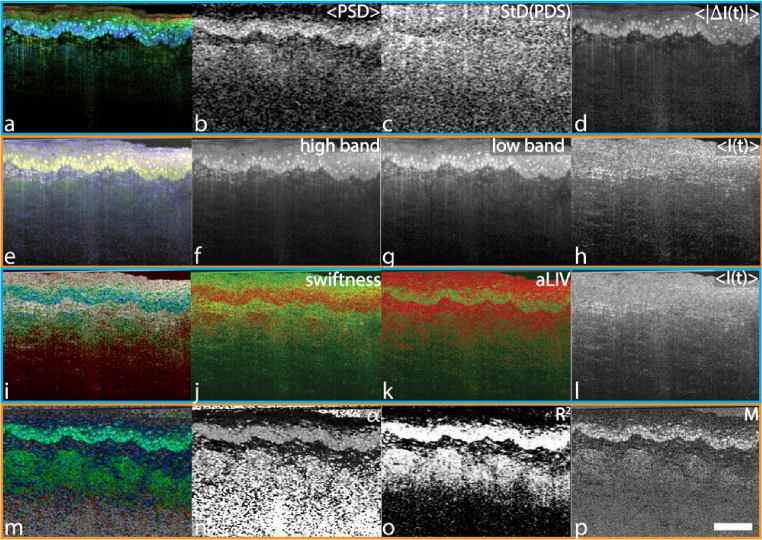
Comparison of the different DOCT algorithms on a mouse tongue explant acquired with the mOCT. The HSB visualization of the PSD moments was computed in 0.44 s. <PSD> ranges from 2.42 to 20.93 Hz, StD(PSD) ranges from 6.53 to 11.78 Hz, and <|ΔI(t)|> ranges from 7.29 to 37.5 dB. The RGB frequency binning was calculated in 1.64 s. The blue channel was determined as the static bin with frequencies < 0.37 Hz, the green channel ranging from > 0.37 to 19.63 Hz, and the red channel > 19.63 Hz. The LIV-based aLIV and Swiftness were computed simultaneously in around 4.37 min. Color image (i) was generated with Swiftness (j) ranging from 5 to 50 s^−1^ for hue (blue-green-red order), aLIV (k) ranging from 0 to 3 dB^2^ for saturation, and OCT intensity (l) ranging from 5 to 30 dB for brightness. The motility-based metrics were computed in 5.47 h. Alpha ranges from 0 to 1.25, $R^{2}$ ranges from 0 to 0.99, and M from 0 to 0.55. Color bars can be found in Supplement 1, Table S1. Scale bar: 100 μm. The full resolution figure can be found in Visualization 3.

**Fig. 4.**
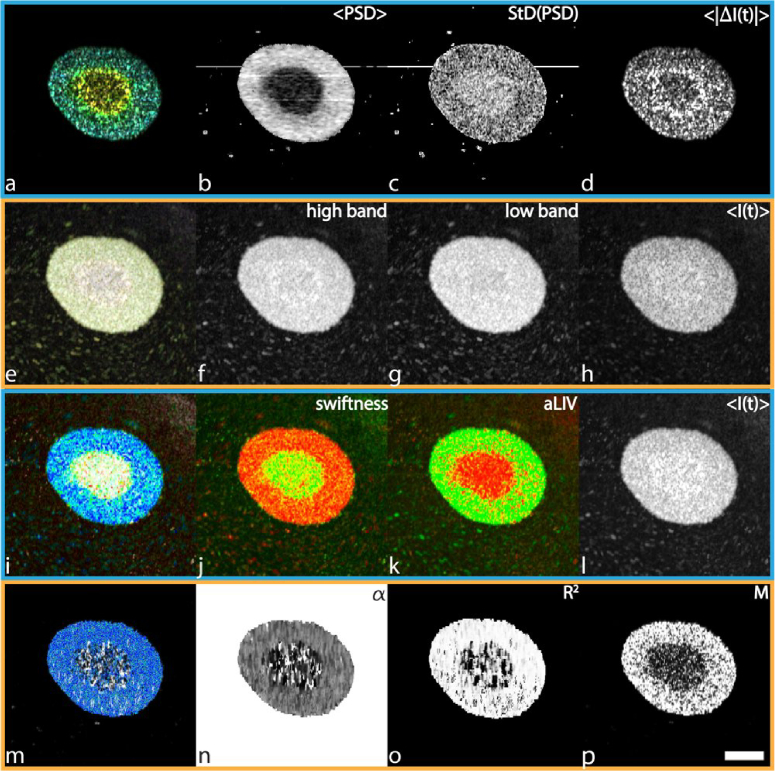
Comparison of the different DOCT algorithms on a untreated MCF-7 spheroid acquired with the JM-OCT. The HSB visualization of the PSD moments was calculated in 0.091 s. <PSD> ranges from 0.024 to 0.145 Hz, StD(PSD) ranges from 0.053 to 0.093 Hz, and <|ΔI(t)|> ranges from 14.26 to 105.0 dB. The RGB frequency binning was calculated in 0.24 s. The blue channel was determined as the static bin with frequencies < 0.08 Hz, the green channel ranging from > 0.08 to 1.14 Hz, and the red channel > 1.14 Hz. The LIV-based aLIV and Swiftness were computed simultaneously in around 6.38 min. Color image (i) was generated with Swiftness (j) ranging from 0 to 3 s^−1^ for hue (blue-green-red order), aLIV (k) ranging from 0 to 5 dB^2^ for saturation, and OCT intensity (l) ranging from 10 to 40 dB for brightness. The motility-based metrics were computed in 2.2 h. Alpha ranges from 0 to 2.2, $R^{2}$ ranges from 0 to 0.98, and M from 0 to 2.01. Color bars can be found in Supplement 1, Table S1. Scale bar: 200 μm. The full resolution figure can be found in Visualization 4.

**Fig. 5.**
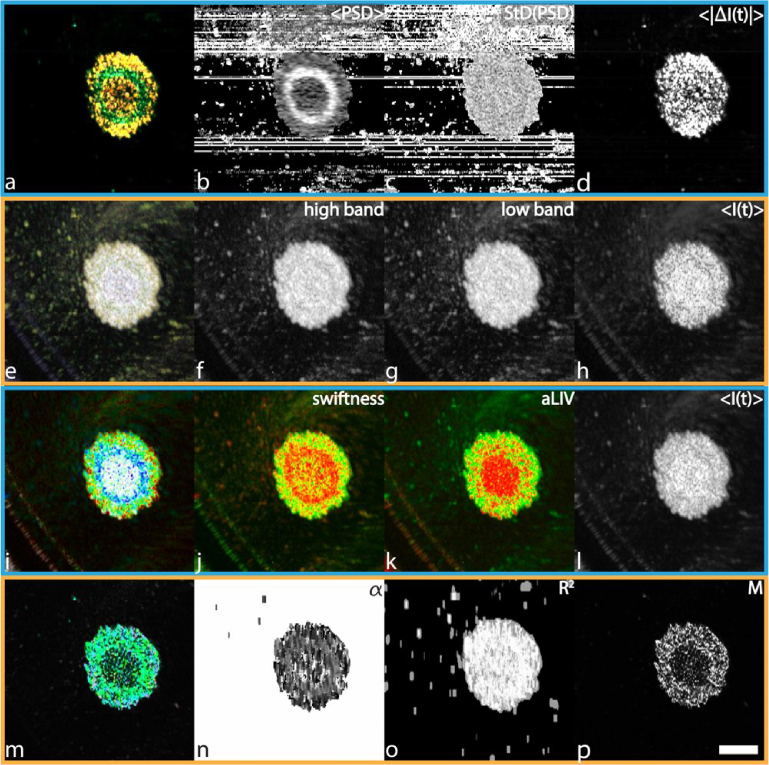
Comparison of the different DOCT algorithms on a MCF-7 spheroid treated with PTX for 3 days acquired with the JM-OCT. The HSB visualization of the PSD moments was computed in 0.091 s. <PSD> ranges from 0.006 to 0.158 Hz, StD(PSD) ranges from 0.052 to 0.095 Hz, and <|ΔI(t)|> ranges from 10.5 to 97.6 dB. The RGB frequency binning was calculated in 0.18 s. The blue channel was determined as the static bin with frequencies < 0.08 Hz, the green channel ranging from > 0.08 to 1.14 Hz, and the red channel > 1.14 Hz. LIV-based aLIV and Swiftness were calculated simultaneously in around 6.38 min. Color image (i) was generated with Swiftness (j) ranging from 0 to 3 s^−1^ for hue (blue-green-red order), aLIV (k) ranging from 0 to 5 dB^2^ for saturation, and OCT intensity (l) ranging from 10 to 40 dB for brightness. The motility-based metrics were computed in 2.2 h. Alpha ranges from 0 to 3.16, $R^{2}$ ranges from 0 to 0.98, and M from 0 to 2.55. Color bars can be found in Supplement 1, Table S1. Scale bar: 200 μm. The full resolution figure can be found in Visualization 5.

For the DFFOCT data (Fig. 1), aLIV and Swiftness were computed using 64 frames extracted at 8-frame intervals from the full 512-frame time sequence, due to PC memory limitations. For the mOCT and JM-OCT data (Figs. 2–5), aLIV and Swiftness were computed using the full time-sequential frames.

The leftmost image (i) in Figs. 1–5 was generated by fusing aLIV and Swiftness. For the DFFOCT data (Fig. 1), Swiftness and aLIV were respectively assigned to hue (ranging from blue to green to red) and brightness, while saturation was fixed at its maximum value. For the mOCT and JM-OCT data (Figs. 2–5), Swiftness, aLIV, and dB-scaled OCT intensity were assigned to hue (blue to green to red), saturation, and brightness. The individual aLIV and Swiftness images are visualized by assigning aLIV or Swiftness to hue (from red to green) and dB-scaled OCT intensity to brightness, following the conventional visualization method.

The LIV-based algorithms were calculated for all samples by Rion Morishita. The respective computer utilized an i7-10750H CPU (2.6 GHz) (Intel), 32 GB of RAM (2.933 GHz), a 512 GB NVMe PCIe 3.0 M.2 2280 SSD (Samsung), and a GeForce RTX 2070 Super with Max-Q Design GPU (NVIDIA).

### 4.4. Motility-based metrics

Three metrics are classically calculated in North Carolina. The first, the motility amplitude, based on a modified standard deviation, M, was calculated as follows: $M(i,j)=\sqrt{\Gamma_{I}(i,j,\Delta t)-{<I(i,j)>}^{2}}/<I(i,j)>$ , with i and j the spatial dimensions (x, y), for the *en face* dataset, and x, z for the B-scan, $\Gamma_{I}(i,j,\Delta t)$ the temporal autocorrelation of I(i,j,t) , Δt is the image sampling time, and thus $\Gamma_{I}(i,j,\Delta t)=\frac{1}{N-1}\sum_{k=1}^{N-1}I(t_{k})I(t_{k+1})$ , as described in [34]. This coefficient is advantageously almost independent of the SNR, as long as the SNR is above 1.

The speckle fluctuation PSD at each image pixel was computed from the discrete Fourier transform. This hyperspectral data was then spatially averaged over the 8 neighboring pixel, and the f≤0 terms were omitted before model fitting. Non-linear least-squares fitting was used with weights, to fit an inverse power law model i.e. $PSD(f)=c_{0}f^{-\alpha }+b_{0}$ , where the free-fit parameters were taken to be the exponent *α*, a constant of proportionality $c_{0}$ , and additive white noise $b_{0}$ , all of which were similarly constrained to be ≥0 during the fitting procedure. The inverse-power-law exponent *α* and the regression $R^{2}$ values were extracted and used as metrics. The inverse-power-law model has been used to describe self-organized critical systems and quantify cellular motility [34], unlike the Lorentzian model, which is known to represent Brownian (diffusive) motions in OCT data [51]. Despite successfully employing these metrics to characterize samples, the underlying meanings of these models remain unclear for biological samples. Nevertheless, a high value of $R^{2}$ , i.e. > 0.95, seems to indicate cellular motility behavior, as it is reduced for fixed samples. *α* provides motility characterization capable of distinguishing e.g. low-to-high-density stromal cells. Further, a low value of *α*, i.e. 0, indicates that the spectrum is noise dominated. We note that *α* is advantageously almost independent of the SNR too, as long as the SNR is above 1. In practice, on the data set used in this work, we had to average the 8 neighboring pixels to obtain an image with sufficient SNR, reducing noise and producing meaningful results.

These three metrics were allocated to an HSB space for rendering purposes, with *α* allocated to the hue channel, $R^{2}$ to the saturation channel, and M to the brightness channel. Manual thresholds were applied, as percentile thresholding did not produce meaningful image rendering in all cases. A GUI for interactive thresholding and rendering was used for the occasion [57]. All the metrics were calculated with MATLAB. M was calculated with a loop on the CPU, which is not a fast implementation of the algorithm but was typically computed in less than 1 s overall. *α* and $R^{2}$ were also calculated in a loop on the CPU, where sequential fitting was performed, resulting in hours of computation time. We note that there might be more optimal implementations of this code, such as parallel fitting on a GPU, but this was not possible in MATLAB, on which these metrics were computed.

The motility-based metrics were calculated for all samples by Tual Monfort. The respective laptop utilized an i7-13850HX (2.1 GHz) (Intel), 64 GB of RAM ThinkPad (Lenovo), a PCIe 4.0 x4 (Lenovo), and an RTX 3500 Ada Generation Laptop GPU (NVIDIA) with 23.04 TFLOPS.

## 5. Results

The aim of this paper is to compare four main DOCT algorithms by analyzing data, which was generated on several OCT instruments available in our research groups. All figures are organized similarly, with the HSB visualization of PSD moments, RGB visualization of frequency bands, LIV-based metrics, and motility-based metrics respectively displayed on the first, second, third, and fourth rows in Figs. 1–5. In order to provide a qualitative comparison between samples, we chose to display the data in a homogeneous manner combining 3 metrics (see section 4) specific to each algorithm processed into a color image (1st column). The independent channels, are displayed in columns 2 to 4 respectively. The metrics of the PSD moments, LIV-based, and motility-based metrics correspond to the hue, saturation, and brightness channels respectively, composing an HSB image. In the case of the RGB frequency binning, the channels correspond to red, green, and blue, yielding an RGB image. It is to note, that the motility-based metrics were not designed to form a combined color image by its authors, and other combinations are very well possible. These visualizations were created from raw data collected from macaque retinal explants acquired by DFFOCT (Fig. 1), mice trachea and tongue explants acquired by mOCT (Figs. 2 and 3), and two MCF-7 spheroids (control and treated with PTX) acquired by JM-OCT (Figs. 4 and 5). For the sake of visualization the metric ranges are only noted in the captions and not displayed as color bars, though they can be looked up in Supplement 1, Table S1. The resulting images are compared based on their ability to accurately distinguish known sample structures. For this, each of these results is first described by the research group who acquired the data, and their observations are then validated by the other teams. An overview of the respective imaging parameters can be seen in Table 1.

**Table 1.** Overview of acquisition parameters by sample

sample	Retina	Trachea	Tongue	Spheroid
system	DFFOCT	mOCT	mOCT	JM-OCT
lateral px spacing	132 nm	1 μm	1 μm	1.95/7.81 μm (x/y)
axial px spacing	N/A	0.62 μm	0.62 μm	7.24 μm
frame rate	100 Hz	108 Hz	111 Hz	4.9 Hz
# Frames	512	150	150	32
acquisition time	5.12 s	1.39 s	1.35 s	6.53 s

### 5.1. Retina

The macaque retinal explant cross-sectional slice, acquired with DFFOCT, displays several retinal layers due to the mounting in a lateral orientation [52]. From the HSB visualization of PSD moments (Fig. 1(a)-(d)), this structural organization of the retina can be easily retrieved, showing, from left to right, photoreceptor outer segments (long blue extensions) and inner segments (green/blue conic shapes), the external limiting membrane (straight black line), and the outer nuclear layer (ONL) filled with photoreceptor nuclei, then the outer plexiform layer, the inner nuclear layer (INL) in which several cell types can be observed, and finally the inner plexiform layer on the rightmost edge. One interesting feature of this sample is that all the different retinal structures display not only different morphological characteristics but also different colors (a). For instance, three cell types can be observed in the INL: large, green cells with circular nuclei, putative horizontal cells, elongated blue cells with high nucleus-to-cytoplasmic ratio, putative bipolar cells, and larger, intense red cells, putative amacrine cells, which are hardly distinguishable in individual channels. An interesting feature of the HSB visualization of PSD moments, and the reason it was made this way, is that the three metrics look significantly different and carry possibly orthogonal information. It is interesting to note that the brightness channel (d) carries most of the high spatial frequencies, while the hue channel (b) can more easily distinguish cell regions from static regions (higher values of hue, which we inverted so as to match low frequencies to cold colors). The photoreceptor outer segments also display a specific high signal (lowest frequencies). It is also interesting to note that in the saturation channel (c), these outer segments look more similar to plexiform regions, which display low saturation, corresponding to more random fluctuations as in cellular regions. This suggests that the plexiform region fluctuates at a low but particular frequency, which corresponds to a particular biological process.

From the RGB frequency binning, specially tailored for the occasion on *en face* data acquired with DFFOCT, we can first comment that the color image looks fairly similar to the HSB visualization of PSD moments, although the color display is different. Similar cell types can be distinguished in the color image (e). However, in this dataset, the three individual channels (f-h) show a higher degree of similarity and redundancy. This can be explained by the fact that in DFFOCT, fluctuations spread over large frequency bandwidths so that cell-specific fluctuations are captured by the intensity ratio of the three frequency channels of the RGB binning. However, the respective ratio of these frequencies are significantly different, which translates into distinguishable structures in the color image.

With the LIV-based processing, the color image (i) enables easy separation of active cell regions from static regions, which was the main purpose of the algorithm. However, the contrast within these regions appears to be lower than for the other algorithms, resulting from it not being designed for single-cell imaging. Furthermore, in the DFFOCT data, <I(t)> does not contain meaningful information. Hence, as described in section 4.3 <I(t)> could not be used in the rendering of the HSB visualization, as it would likely just degrade the HSB image contrast. Similarly, it was not used as brightness in the display of the swiftness and aLIV metrics, which are displayed in grayscale instead. Regarding these individual channels (j-l), it is interesting to see that the different cell types are distinguishable in the aLIV (l), while the Swiftness (j) displays a specific black signature for a few other cells (which stand out in (a) and (e) as well).

Regarding the motility-based metrics (m-p), the color image, as well as the individual channels mostly show a difference between static and dynamic structures.

### 5.2. Trachea

Histology of the mouse trachea sample [47] which features in the mOCT image shows that it consists of connective tissue, which is expected to show low activity, above which an epithelial layer resides, of medium to fast dynamics. On top is a ciliary layer of high frequency activity. The connective tissue further contains several individual cells. Indeed, DOCT with the RGB frequency binning, clearly shows these three distinct layers in mouse trachea (Fig. 2(e)-(h)): the cilia on top (yellow), the epithelium in the middle (green), and the connective tissue below (blue/purple). Further, the connective tissue contains immune cells (green) as well as large chondrocytes within the cartilage region (yellow/green, right side). The purple color within the connective tissue most likely results from the combination of the static tissue with vibrational system noise, as the average spectrum of the sample unveils distinct peaks around 20 Hz (data not shown).

With the HSB visualization of the PSD moments (a-d), the cilia are even better distinguishable. The epithelium and connective tissue are distinguishable as well, though this is slightly harder, as both have a similar greenish color, likely due to the vibrational noise on top of the static tissue and the static information being discarded in this processing scheme. The immune and cartilage cells are also clearly visible. Additional orange spots of unknown origin appear in the upper left region of the epithelium and could be additional cellular activity. Furthermore, the sample is thin enough for the signal decrease, which depends on the distance to the focal plane, to not inhibit the results of the PSD moments algorithm.

With the LIV-based processing (i-l), the cilia, epithelium, and connective tissue can be differentiated within all metrics. Swiftness (j), however, is highly affected by the vibrational noise and therefore displays the static connective tissue as very dynamic. The cellular structures are visible with all metrics, though they are a lot harder to see due to diffuse borders. In particular, the cartilage cells are hard to distinguish from the immune cells.

With the motility-based approach (m-p), the ciliary layer and epithelium are hard to distinguish in any of the metrics. The connective tissue is well visible within the M metric (p) together with cartilage cells. The immune cells remain partly hidden by noise.

### 5.3. Tongue

The murine tongue which features in the mOCT image shows a composition of five different layers in Histology [38]. At the top is stratum corneum, which should exhibit low frequencies, followed by an underlying epithelium with a granular layer of higher activity. Beneath it resides the basal layer with very active cells. Further below is connective tissue, expected to be rather static, together with skeletal muscle fibers of medium activity. The RGB frequency binning (Fig. 3(e)-(h)) can distinguish stratum corneum with keratinized cells on top (blue, purple) from the underlying epithelial layer (light pink/yellow) and the basal layer with single cell nuclei visible inside (green, yellow). Further, connective tissue (blue, purple) with skeletal muscle fibers (green) are well observable. The purple coloring once again is caused by vibrational system noise.

When using HSB processing to visualize the PSD moments (a-d), the dependency of the signal strength is noticeable, as dynamic information vanishes for regions further away from the focal plane. In contrast, the stratum corneum (dark red) seems better differentiated. Moreover, the algorithm offers a higher dynamic range for the granular layer (green) and the basal layer (blue), giving more insight into cell activities. Additionally, cell boundaries are observable around the bright nuclei, a feature not observable with other approaches. The connective tissue and skeletal muscle fibers seem equally differentiable.

In the LIV-based processing (i-l), the stratum corneum cannot be differentiated from the granular layer. The single nuclei in the basal layer are visible in all metrics, though borders are not as sharp. The underlying connective tissue and fibers are visible as well, though better in the Swiftness (j) than in the aLIV (k) and Fusion (i) metrics. However, the Swiftness (j) metric is strongly affected by the vibrational noise again, yielding high dynamics for the otherwise static tissues.

In the motility-based metrics (m-p) the stratum corneum cannot be distinguished from the granular layer either. Further, in $R^{2}$ it cannot be distinguished from the background. The single nuclei on the other hand are well visible within the M (p) and fusion (m), though the resolution seems inferior to the HSB visualization of the PSD moments (a) and the RGB frequency binning (e). Within the other metrics, single nuclei cannot be seen at all, only appearing as a continuous layer, possibly due to the averaging with the 8 neighboring pixel. The connective tissue is visible as well, with the muscle fibers more easily observable in the fusion (m) than with any of the other groups metrics, likely due to their strong representation in the $R^{2}$ (o) and M (p) metrics.

### 5.4. Spheroid

The spheroids, imaged with JM-OCT, display circular layers of various activity. Fluorescence imaging of the untreated (control) MCF-7 spheroid highlighted a concentric pattern consisting of two distinct regions, where the inner and peripheral regions corresponded to dead and living cells, respectively [26]. In the color-fusion image from the LIV-based metrics (Fig. 4(i)-(l)), the spheroid exhibits two concentric layers with distinct dynamics, as well. The central region appears white due to low aLIV, and it suggests the low occupancy of moving scatterers. In the Swiftness image (j), the central region also shows high values, i.e. fast movement of scatterers. In contrast, the peripheral region appears as a mix of blue and light blue, corresponding to a combination of high aLIV (k), suggesting a high occupancy, and relatively low Swiftness (j), indicating slow changes. These two concentric layers may correspond to the well-known necrotic core and vital periphery of tumor spheroids, respectively [53,54]. Around the spheroid one can see particles floating, respectively with high swiftness and aLIV.

Using the HSB visualization of the PSD moments (a-d), the spheroid exhibits three distinct layers: a high-frequency innermost core (yellow), a moderate-frequency ring (green), and a low-frequency peripheral region (a mix of blue and light blue). The innermost and outermost layers may correspond to the necrotic core and vital periphery, also observed in the LIV-based image. The moderate-frequency ring may correspond to a quiescent layer between the necrotic core and the vital periphery [54,55]. The floating particles become invisible within the HSB image due to their absence in the brightness channel (d).

The RGB frequency binning algorithm (e-h) reveals two layers, namely, necrotic core and vital periphery, similar to those in the LIV-based image. The necrotic core appears white indicating a mixture of three frequency bands, while the vital periphery appears green indicating a moderate frequency. However, the contrast is only faint. Furthermore, the particles become extremely visible with this algorithm, especially due to their strong presence within the high (f) and low bands (g).

The motility-based approach (m-p) also highlights the necrotic core. In the combination image (m) it exhibits a tessellated appearance with white and black spots, while the vital periphery exhibits a blue color. The tessellated appearance in the core is attributed to low M (p) and low $R^{2}$ (o), while the blue color in the periphery corresponds to high M (p) and moderate *α* (n). Notably, M (p) shows a similar contrast to aLIV (j), as both metrics measure the magnitude of OCT signal fluctuations. The particles become invisible due to their absence in any of the channels.

The fluorescence image of the spheroid treated with PTX for 3 days demonstrated that the living and dead cells are intermixed [26]. Using the LIV-based approach (Fig. 5(i)-(l)), the spheroid exhibited three concentric layers with different dynamics. In the fusion image (i) the central and intermediate layers appear white and in a mixture of blue and light blue, which are consistent with the appearances of the necrotic core and vital periphery of the untreated spheroid. The outermost layer appears green indicating high aLIV (k), due to a high occupancy of moving scatterers, and moderate swiftness (j), corresponding to them moving at moderate speed. In addition, several red spots are visible in the outermost layer, which correspond to high aLIV (k), thus high occupancy, and high swiftness (j), caused by fast signal fluctuations. Once again floating particles can be observed with high swiftness (j) and aLIV (k), but near vanish in their combined image (i).

The central and intermediate layers in the HSB visualization of the PSD moments (a-d) and RGB frequency binning images (e-h) also exhibit appearances consistent with the necrotic core and the vital periphery of the untreated spheroid in their respective images. In both the HSB visualization of the PSD moments (a) and the RGB frequency binning image (e), the outermost layer appears yellow, indicating medium to high frequencies. However, the RGB frequency binning offers less contrast within the spheroid. The floating particles again cannot be seen within the HSB visualization of the PSD moments, but are even more prominent within the RGB frequency binning.

It should be noted that there are several horizontal line artifacts visible in Fig. 5(b)-(c). These have been caused by normalization during the contrast adjustment, which was performed on each B-scan individually.

The result from the motility-based approach (m-p) also reveals three concentric layers. While the central and intermediate layers of the treated spheroid resemble the necrotic core and vital periphery of the control spheroid in the other algorithms, the motility-based algorithm shows different appearances between these layers in the treated and untreated spheroids. Specifically, in the HSB image (m) the central and intermediate layers of the treated spheroid appear black and green, while the necrotic core and the vital periphery of the untreated spheroid appeared as a tesselation of black and white, and blue color, respectively. The innermost layer (almost black) corresponds to low M (p), the intermediate layer (green) corresponds to a combination of high M (p) and moderate *α* (n), and the outermost layer (blue) corresponds to a combination of high M (p) and low *α* (n). The particles are not visible once again.

## 6. Discussion & conclusion

As shown in the results, each of the algorithms has its benefits but also its shortcomings. This is likely due to each processing being tailored to its own typical acquisition and samples. We noticed several key aspects.

In the HSB visualization of the PSD moments, the discarding of the static component benefits them, as it offers a larger dynamic range for the actual dynamic properties of the sample, yielding a higher possibility to differentiate activity, such as for different cell types or the different layers of the spheroids. On the other hand, it somewhat increases their dependency on a good SNR, which is not an issue in *en face* planes but slightly limits their capabilities in out of focus areas when applied to B-Scans.

The RGB frequency binning algorithm on the other hand uses the static information and thus sacrifices the higher dynamic range to gain structural sample information even for areas of vanishing signal strength. Interestingly one can further observe in these results that the approach seems to be very sensitive to resolvable motion, such as within the DFFOCT and mOCT data where even small cells can clearly be observed, but being severely limited on data where all dynamic processes happen below the optical resolution. Hence, the contrast on the JM-OCT data is clearly inferior to the other algorithms, only barely being able to distinguish the core and peripheral regions.

On the contrary, the LIV-based metrics shine within this domain, yielding great results for sub-resolution dynamics. When moving to the high resolution imaging the metrics produce good results for the DFFOCT data as well, but start to struggle with the mOCT data, which contains vibrational system noise. Here, the aLIV seems rather resilient against the noise, but the swiftness is highly affected, displaying the static tissues as very active. The fusion combination of the two metrics circumvents this noise dependency of the Swiftness. However, all the LIV-based metrics have difficulties nonetheless to sharply differentiate signals in these noise-affected images, where small structures like e.g. cells often have diffuse boundaries.

For the motility-based metrics the results also vary greatly. While the M metric always seems to produce good results, accurately capturing fine details and being very resilient against poor SNR, the other metrics seem to produce inferior results, resulting in highly varying quality of their combination. However, it is especially important to note that while the combined color images did not contain a dedicated structural channel, this entire functional image nonetheless yielded great results on the tongue and spheroid data, only struggling slightly with tissue distinguishability on the retina and trachea data.

Some of the DOCT algorithms are sensitive to the magnitude of signal fluctuation, and some others are sensitive to the speed of the signal fluctuation. LIV, aLIV, and M are examples of former, where LIV and aLIV are the time variance in the dB-scaled (i.e., logarithmic) intensity, while M is the contrast of signal fluctuation in the linear intensity scale. Both Swiftness and *α* are examples of the latter. However, Swiftness is a metric computed from time statistics of the OCT signal, while *α* is computed from PSD. This essential difference can cause different contrast patterns between two algorithms.

Further, it is to note that while the DOCT images often appear granular in appearance, this is not to be confused with speckles. Speckles are an inevitable phenomenon of OCT imaging, and are the carrier of dynamic information in DOCT. DOCT extracts the tissue dynamics by analyzing the temporal properties of the speckle. Therefore, the granular appearance in the DOCT images is not the speckle pattern itself, but an effect caused by it in the original OCT data. When the total sampling duration is sufficiently long, the OCT signal at a specific point fully alternates between dark and bright speckles. In this case, the DOCT signal becomes insensitive to the state of the original speckle (i.e., dark or bright) and hence, the granular appearance of DOCT may become less pronounced. On the other hand, when the sampling duration is short in respect to the OCT signal fluctuation speed, or similar to the speed of the sample dynamics, the DOCT value can be affected by the original state of the speckle, which may cause granular, i.e., speckle-like, appearance. However, the relationship between the OCT speckle and the granular appearance is complicated and depends on the DOCT algorithm. It may be an important future work to investigate this point, for example by using numerical simulations of DOCT [56], but is outside the scope of this publication.

Overall it became quite clear that there currently is no algorithm who outperforms the rest in all scenarios. Instead all of the algorithms clearly show their strengths mainly with the images of the group they were designed by. Areas to improve become obvious on foreign data. Hence, each routine remains tied to one optimal use case, mainly depending on the available imaging setup. It is important for any user to be aware of their pros and cons to be able to correctly interpret their results. Future work should thus try to incorporate the advantages of each to try and combine them into one, offering dynamic contrast that is as independent as possible of the imaging parameters used.

## Supplemental information

Supplement 1 Supplement 1 containing the average OCT Intensity of the DFFOCT Retina data and the supplementary color bars for all figures. https://doi.org/10.6084/m9.figshare.30271783

Visualization 1 Full Resolution version of Fig. 1. https://doi.org/10.6084/m9.figshare.30218242

Visualization 2 Full Resolution version of Fig. 2. https://doi.org/10.6084/m9.figshare.30218254

Visualization 3 Full Resolution version of Fig. 3. https://doi.org/10.6084/m9.figshare.30218257

Visualization 4 Full Resolution version of Fig. 4. https://doi.org/10.6084/m9.figshare.30218251

Visualization 5 Full Resolution version of Fig. 5. https://doi.org/10.6084/m9.figshare.30218260

## Data Availability

Data underlying the results presented in this paper are not publicly available at this time but may be obtained from the authors upon reasonable request. Code is available at [57].

## Supplemental document

See Supplement 1 for supporting content.
