# Supplementary material for: Guide to dynamic OCT data analysis
Source: Biomed Opt Express. 2025 Oct 31;16(11):4851–70. doi: 10.1364/BOE.571394 (PMC12642996; doi:10.1364/BOE.571394)
Supplement: Supplementary file 1 [file boe-16-11-4851-s001.pdf]

Supplemental document accompanying submission to *Biomedical Optics Express*

**Title:** A guide to dynamic OCT data analysis

**Authors:** Noah Heldt, Tual Monfort, Rion Morishita, Robert Schönherr, Olivier Thouvenin, Ibrahim El-Sadek, Peter König, Gereon Hüttmann, Kate Grieve, Yoshiaki Yasuno

**Submitted:** 7/4/2025 5:31:10 PM

OPTICA
PUBLISHING GROUP

# A guide to dynamic OCT data analysis: supplemental document

## DFFOCT INTENSITY

Supplemental Figure S1 to showcase that the average intensity could not be used for the RGB frequency binning and LIV based algorithms, as it does not contain meaningful, structural information due to the nature of the acquisition.

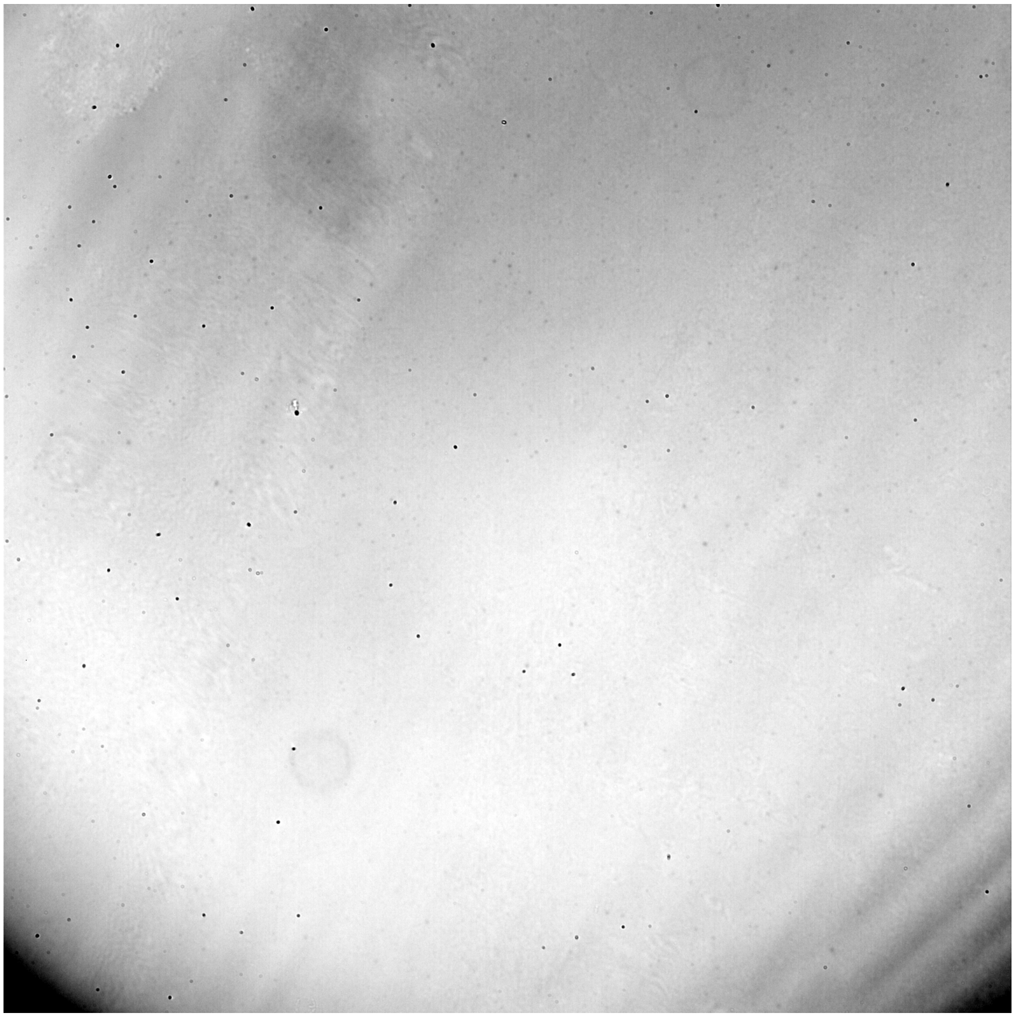

**Fig. S1.** Average OCT Intensity of the DFFOCT Retina data. As seen, it does not contain meaningful, structural information due to the nature of the acquisition.

## EXTENDED COMPARISON WITH MEAN AND STANDARD DEVIATION METRICS

Supplemental table S1 displaying the color bars of the respective metrics

**Table S1.** Color bars for the figures.

| Figure | 1: Retina | 2: Trachea | 3: Tongue | 4: Spheroid control | 5: Spheroid day 3 |
|---|---|---|---|---|---|
| PSD | 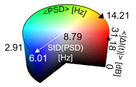 | 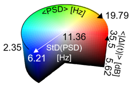 | 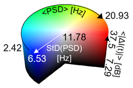 | 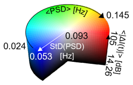 | 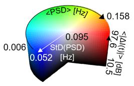 |
| RGB | 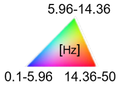 | 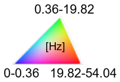 | 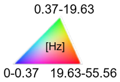 | 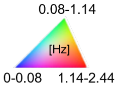 | 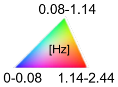 |
| LIV Fusion | 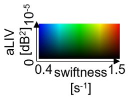 | 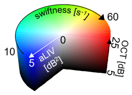 | 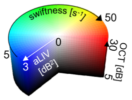 | 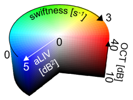 | 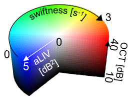 |
| Swiftness | 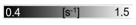 |  |  |  |  |
| aLIV | 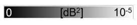 |  |  |  |  |
| OCT | N/A |  |  | 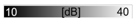 |  |
| Motility Fusion | 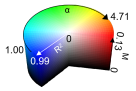 | 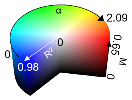 | 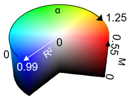 | 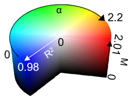 | 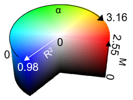 |
