# Supplementary figures and images for: Guide to dynamic OCT data analysis
Source: Biomed Opt Express. 2025 Oct 31;16(11):4851–70. doi: 10.1364/BOE.571394 (PMC12642996; doi:10.1364/BOE.571394)

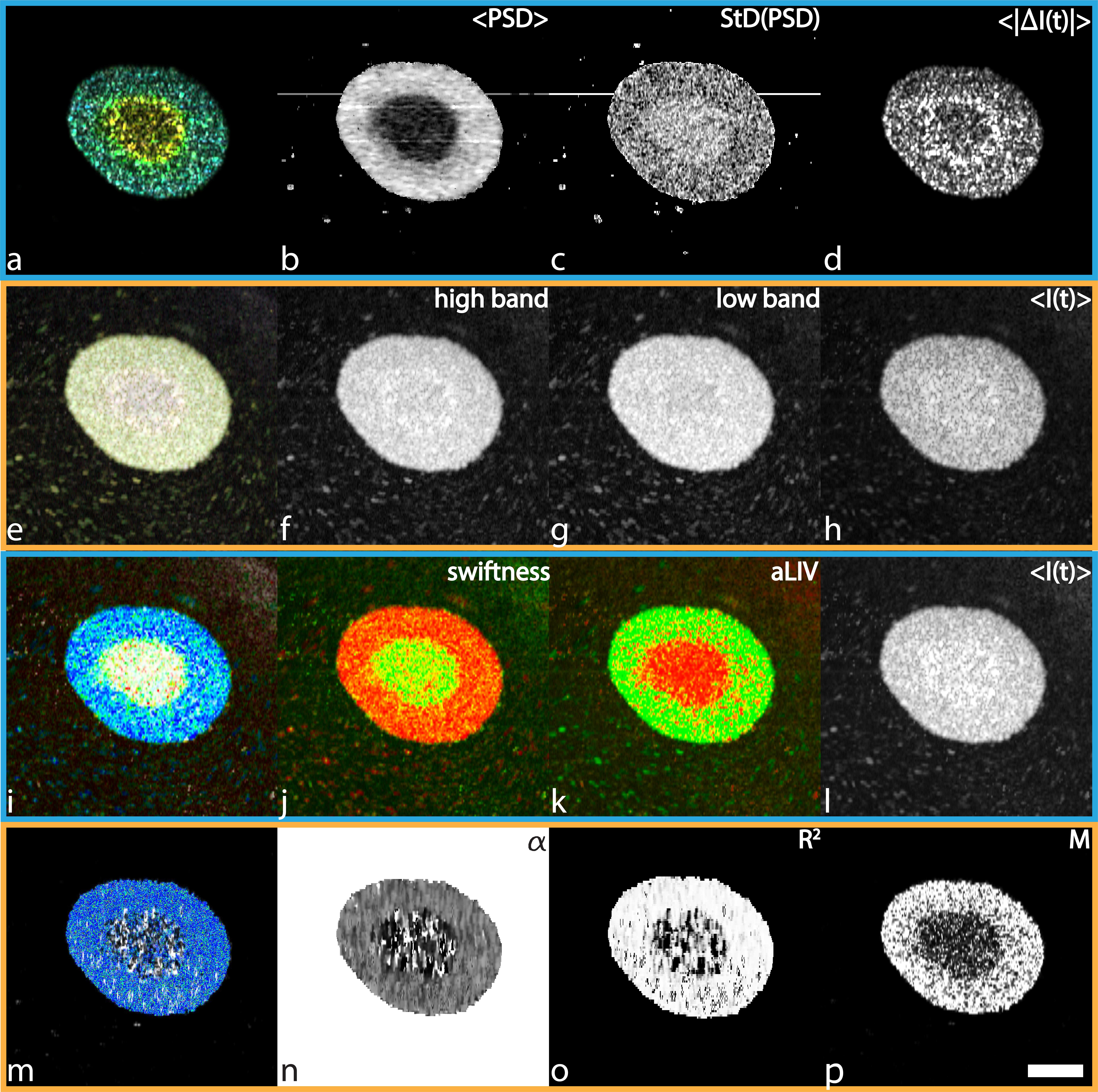

Supplement: Supplementary file 5 [file boe-16-11-4851-v004.jpg]

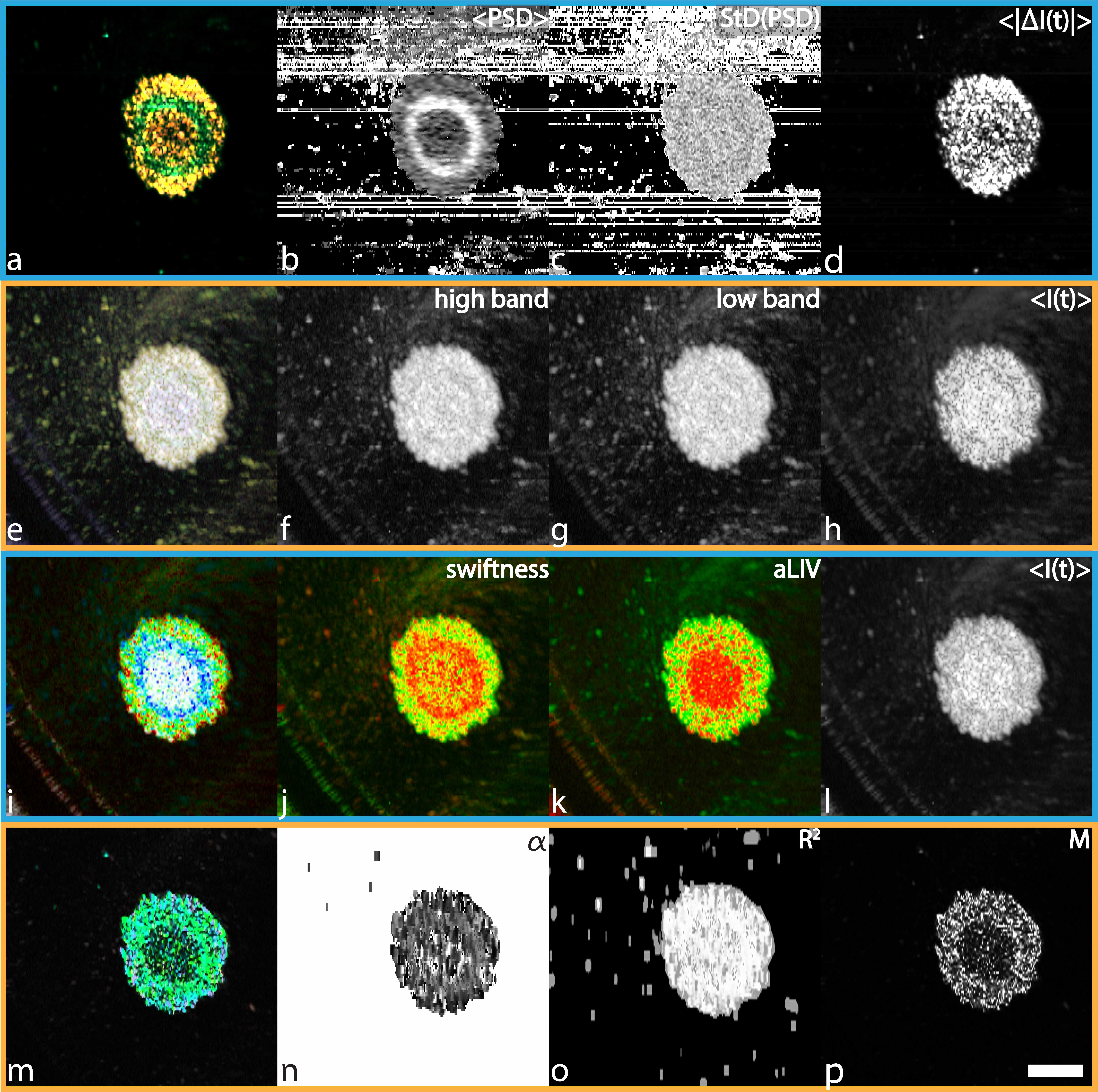

Supplement: Supplementary file 6 [file boe-16-11-4851-v005.jpg]
